# Examining the Effect of Peer-Led Parenting Interventions on the Wellbeing of Parents With Children With an Emotional or Behavioural Disorder – A Systematic Review

**DOI:** 10.1177/13591045261418322

**Published:** 2026-01-26

**Authors:** Meaghan Reitzel, Kayla Brissette, Ledina Hasanagic, Mona Elmikaty, Lori Letts, Briano Di Rezze, Oksana Hlyva, Anne MacLeod, Michelle Phoenix

**Affiliations:** 1 School of Rehabilitation Science, 62703 Faculty of Heath Sciences, McMaster University, Hamilton, ON, Canada; 2 CanChild Centre for Childhood-onset Disability Research, School of Rehabilitation Science, McMaster University, Hamilton, ON, Canada; 3 Bachelor of Health Sciences (Honours) Program, 62703 Faculty of Heath Sciences, McMaster University, Hamilton, ON, Canada; 4 Honours Life Sciences Program, 62703 Faculty of Science, McMaster University, Hamilton, ON, Canada; 5 Department of Pediatrics, 62703 Faculty of Health Sciences, McMaster University, Hamilton, ON, Canada; 6 Parent Partner, Hamilton, ON, Canada; 7 113807 KidsAbility Centre for Child Development, Waterloo, ON, Canada

**Keywords:** systematic review, peer-led interventions, parenting interventions, parent wellbeing, emotional and behavioural disorders

## Abstract

**Background:**

Parents of children with emotional or behavioural disorders (EBD) experience challenges with their own wellbeing. Parenting practices are impacted by parent wellbeing and are vital to supporting children with EBD. Programs with peer-led components, aimed at developing healthy parenting skills have been developed. While peer-led parenting interventions for parents of children with EBD have led to positive outcomes for children, outcomes related to parent wellbeing are not known. This systematic review examines the impact of peer-led parenting programs on the wellbeing of parents of children with EBD.

**Methods:**

A systematic review across 7 databases was conducted. 1009 articles were screened, and 13 met criteria for inclusion.

**Results:**

This study identified 8 peer-led parenting interventions. 11 outcome measures of parent wellbeing were used across the 13 studies. 7 studies reported statistically significant improvement in parent wellbeing outcomes. The 6 studies that did not report statistically significant improvement showed a trend toward improved parent wellbeing on outcome measure scores.

**Conclusions:**

Findings indicate the potential for peer-led parenting interventions to have a positive impact on parent wellbeing. Future research is needed to determine how to meaningfully measure parent wellbeing and what aspects of peer-led parenting interventions positively impact parent wellbeing.

## Plain Language Summary

Emotional or Behavioural Disorders (EBD) are conditions that impact on children’s abilities to manage their emotions, behaviours or social interactions. Parents of children with these difficulties often struggle with their own wellbeing, which can affect how they care for their child. Parenting support programs led by other parents (peers) exist, but their impact on parent wellbeing is unclear. This study reviewed 13 research articles on peer-led parenting programs. Findings indicate the potential for peer-led parenting interventions to have a positive impact on parent well-being. In the articles included, parent wellbeing was measured in different ways and many different peer-led parenting interventions were described. Future research is needed to determine meaningful ways to measure parent wellbeing and to better understand what aspects of peer-led parenting interventions positively impact parent wellbeing.

## Introduction

It is estimated that 20% of Canadian children and youth will experience challenges with their mental health and that 70% of mental health concerns begin in childhood or adolescence (Canadian Mental Health Association, 2025a). Signs of mental health concerns in children and youth can take the form of emotional or behavioural challenges such as avoiding friends or family, changes in sleeping or eating habits, persistent worrying, outbursts of anger, rebelling against authority, engaging in risk-taking behaviours or experiencing regular mood swings (Canadian Mental Health Association, 2025b). In the United States, approximately 21% of children aged 3-17 are diagnosed with an emotional or behavioural disorder (Centers for Disease Control and Prevention, 2025). According to data from 2022-2023, 11% of American children have diagnosed anxiety, 8% are diagnosed with a behavioural condition and 4% are diagnosed with depression (Centers for Disease Control and Prevention, 2025).

The term emotional and behavioral disorders or difficulties (EBD) represents mental health conditions that are not well-defined in the literature. There is a lack of consistent terminology and agreed upon acceptance for what diagnoses fit under the EBD umbrella, which also leads to challenges with obtaining accurate prevalence estimates (Boydell Brauner & Bowers Stephens, 2006; Forness & Kavale, 2000). Although there is notable inconsistency in how EBD are defined and prevalence is reported, there is consensus that untreated childhood EBD can have negative effects on school performance, peer relationships, sleep patterns, and employment prospects (Ogundele, 2018). For the purpose of this review, EBD is conceptualized as an emotional or behavioural disorder, challenge or difficulty that negatively impacts a child or youths’ day-to-day function and participation (Ringeisen et al., 2020). Some conditions commonly described as EBD include mood disorders such as depression, anxiety disorders such as obsessive-compulsive disorders (OCD) and behaviour disorders such as oppositional defiant disorder (ODD) (Ringeisen et al., 2020). EBD prevalence estimates are further complicated by underreporting linked to the underuse of supports available to families of children with an EBD (Ringeisen et al., 2020). Barriers such as stigma for seeking mental health treatment and parent uncertainty about what types of emotional disturbances or behaviours constitute as atypical may make it challenging for families to access available supports, contributing to underuse (Ringeisen et al., 2020). Though reported prevalence of EBD varies greatly within the literature, it is estimated that 20% children in the United States will experience an EBD before the age of 18 (Ringeisen et al., 2020).

EBD in children and youth have been associated with increased parental strain including higher stress levels (Bode et al., 2016; Bögels et al., 2014). High levels of parental stress persist across demographic domains including the child’s age, the child’s gender, and family socio-economic status (Bode et al., 2016). Strain of parenting a child experiencing emotional or behavioural challenges may be increased when parents themselves experience challenges with their mental health (Bögels et al., 2014). In a study examining the psychological distress in parents of children with psychiatric disorders, 54.5% of parents reported experiencing severe psychological distress (Saed O’leimat et al., 2019). Findings of a 2021 scoping review indicate that 1 in 3 parents of children receiving mental health services report experiencing a mental illness (Campbell et al., 2021). Parent mental health challenges can lead to poorer outcomes related to child behavioural and emotional development and mental health interventions (Campbell et al., 2021; Smith, 2004). Further, parenting a child with an EBD can impact how a parent feels they perform in other areas of their lives such as at work and in social relationships (Campbell et al., 2021). In families where both the child and parent experience mental health challenges, capacity for parenting and the parent-child relationship can be impacted (Campbell et al., 2021). Although parents experiencing mental health challenges will often seek support for their children, they less frequently seek support for themselves (Campbell et al., 2021). Given the impact of mental health on parenting practices, screening parent wellbeing and addressing identified concerns has been recommended in the child mental health field (Campbell et al., 2021). Although individual concepts such as parent mental health (Duncombe et al., 2012), psychological distress (Saed O’leimat et al., 2019) and caregiver strain (Mayberry & Heflinger, 2013) have been explored in the EBD literature; parent wellbeing as a general construct has not been clearly defined.

The Conceptual Model of the Factors that Influence Parent Wellbeing is a dynamic process model that provides a framework for explaining factors influencing parent wellbeing by simultaneously considering the impact of personal factors (e.g., family demographics, parent mental health) and environmental factors (e.g., social supports, access to information) (Resch et al., 2012). The model theorizes that outcomes of parent wellbeing are impacted by a parent’s mental/emotional health, physical health, personal life satisfaction and family satisfaction (Resch et al., 2012). In this model, parent wellbeing is influenced by whether parents appraise their environmental/social characteristics (e.g., access to services) and parent and child characteristics (e.g., disability severity) as an opportunity for growth or as a threat (Resch et al., 2012). Although developed for use with parents of children with disabilities, this conceptual model provides a holistic framework for parent wellbeing that can be applied to parents of children with EBD and informed how we defined this construct.

The link between parent mental health (or more broadly positioned as parent wellbeing) and associated parenting practices is of the upmost importance as some parenting practices such as inconsistent discipline have been linked to the development of problematic child behaviours and emotional difficulties (Berg-nielsen et al., 2003; Duncombe et al., 2012). By contrast, parenting practices that are consistent, empathic, and validating of emotions have been demonstrated to have positive outcomes for children (Duncombe et al., 2012). As a result of the impact of parenting practices on outcomes for children with EBD, interventions focusing on parenting practices, beliefs, feelings and the overall child-parent relationship (Scott and Gardner, 2015) are common in a multi-faceted approach to the treatment of EBD. Parenting programs aim to positively impact the parent-child relationship by improving the quality of parenting provided to a child and have been demonstrated to be effective at reducing behavioural challenges in children, decreasing parent stress levels, improving parent mental health outcomes and increasing parents’ feeling of competence (Högström et al., 2017; Scott and Gardner, 2015; Stattin et al., 2015). Two systematic reviews examining the effectiveness of parenting interventions in the EBD population indicated that they had the potential to positively influence outcomes related to behavior and emotional functioning in both younger and older children (Barlow et al., 2005, 2016).

Peer-led programs (i.e. programs run by parents for other parents) have been utilized in many fields including chronic disease self-management (Funnell, 2010), smoking cessation (Apata et al., 2019) and caregiver support (Han et al., 2022). In some health-related fields, the use of peer-led interventions has been found to be effective in knowledge sharing and supporting changes in behaviour, however more evidence is needed to generalize the effectiveness of this intervention approach more broadly (Topping, 2022). In parents of children with disabilities, peer-led interventions aimed at health promoting behaviours in parents have been found to be feasible in community settings (Bjornstad et al., 2021). This model of intervention has been used to support parents of children with emotional and behavioural challenges gain parenting practices that promote positive outcomes for the child and the family (January et al., 2016; Michelson et al., 2014). Further, a peer-led approach to parenting intervention was found to be an acceptable format for parents with self-identified difficulties related to parenting (Bradley et al., 2020). In a peer to peer support program for parents of children with a neurodisability, parents felt understood by their peer supporters and that shared elements of their lived experience helped with developing a genuine connection (McCrossin & Lach, 2022). Further, opportunities to connect with peer supporters, allowed parents to envision a hopeful future for their children (McCrossin & Lach, 2022). This hopeful state, may positively impact overall family satisfaction, a factor described as influencing parent wellbeing in the Conceptual Model of the Factors that Influence Parent Wellbeing (Resch et al., 2012).

Parenting has been demonstrated to impact outcomes for children with EBD and a parent’s sense of wellbeing has been shown to influence parenting practices. It is important to understand if participating in a peer-led parenting intervention has positive outcomes for parents because parenting interventions are a common approach to the treatment of EBD in children. Currently, the impact of participating in peer-led parenting interventions on parent wellbeing is not known in the EBD literature. This systematic review examines the question: For parents of children or youth with emotional and behavioural disorders or difficulties, does participation in peer-led parenting interventions result in improved parent wellbeing?

The objectives of this systematic review are to.

1. Describe the types of peer-led parenting interventions identified in the literature.
2. Describe the range of outcomes measured in the literature related to parent wellbeing when peer-led parenting interventions are delivered.
3. Identify if participation in peer-led parenting interventions is beneficial for the wellbeing of parents of children or youth with EBD.
4. Make recommendations about the use of peer-led parenting interventions in the population of parents of children and youth with EBD and areas for future research.

## Methods

This systematic review was completed in alignment with the protocol registered with the National Institute for Health Research international prospective register of systematic reviews (PROSPERO CRD42022300596) (Reitzel et al., 2022) and is reported according to the updated Preferred Reporting Items for Systematic Reviews and Meta-Analysis (PRISMA) statement (Page et al., 2021) and the Synthesis Without Meta-Analysis (SWIM) reporting guidelines (Campbell et al., 2020). Deviations from the protocol are reported in the methods section of this paper.

### Development of the Research Question and Objectives

After reviewing the pediatric EBD literature, our research question and objectives for this systematic review were developed by an interdisciplinary team. Our team includes rehabilitation science researchers with clinical backgrounds in occupational therapy and speech and language pathology and two parent partners with lived experience of having a child with an EBD. The team is located at McMaster University in Ontario, Canada. Based on feedback from our parent partners about the research question and objectives, the word “difficulty” was used in addition to disorder to be inclusive of children and youth who may experience emotional and behavioural challenges but who do not have a diagnosed disorder.

### Identification and Selection of Studies

The search strategy was developed in consultation with an experienced health sciences librarian. Electronic searches of the Web of Science, Education Resources Information Centre (ERIC), Cumulative Index to Nursing and Allied Health Literature (CINAHL) and MEDLINE, EMBASE, EMCare and PsychINFO via OVID were completed in December 2021 and updated in September 2024. No limits were placed on language, publication period or publication type for all databases with the exception of CINAHL. Under advisement from the research librarian, the search in CINAHL was restricted to peer reviewed literature. Grey literature and unpublished studies were not searched. The search strategy was developed using synonyms of four core concepts of the review: parents (e.g., mother, father), parenting intervention (e.g., parenting program), peer-led (e.g., peer-facilitated) and EBD (e.g., depression). A comprehensive list of search terms is provided in the Supplementary Material. Covidence was used to collate and deduplicate search yields (Veritas Health Innovation, n.d). Duplicates removed by Covidence were reviewed for accuracy and additional duplicates were removed and tracked manually during title and abstract screening. The references of articles included after full-text screening were searched for additional relevant primary studies.

Review of the literature and discussions with the review team guided development of the inclusion and exclusion criteria. Parent partners provided feedback on the inclusion and exclusion criteria which helped to establish an inclusive definition of the construct of parent and finalize the EBD to be included. The inclusion and exclusion criteria were piloted by two reviewers (Reviewer 1, Reviewer 2) through independently screening a small sample of articles, comparing decisions, discussing disagreements and making adjustments to the criteria accordingly.

Studies that met inclusion criteria were peer-reviewed articles published in English and included the following criteria: (a) parents (including stepparents, adoptive parents, foster parents, mothers and fathers) of a child with an EBD aged 0-18 years; (b) focused on children or youth with depression, dysthymia, bipolar disorder, anxiety disorder, agoraphobia, separation anxiety disorder, social anxiety disorder, post-traumatic stress disorder, obsessive-compulsive disorder, specific phobia, panic disorder, attention deficit with hyperactivity disorder, attention deficit disorder, oppositional defiant disorder, conduct disorder, eating disorders or generalized emotional or behavioural disorders or difficulties. Included EBD were determined according to epidemiological literature exploring the prevalence of common EBD (Ringeisen et al., 2020; Waddell et al., 2014); (c) contained a peer-led (could be co-led with parent peer and clinician) individual intervention or group-based parenting intervention; (d) had an outcome related to parent wellbeing extending beyond measuring change in parenting skills or knowledge. Wellbeing was defined using the Conceptual Model of Factors Influencing Parent Wellbeing by Resch et al. (2012). Aligned with this model, studies needed to have an outcome measure broadly related to parent mental/emotional health, physical health, personal life satisfaction or family satisfaction to be included (Resch et al., 2012). Given the interest in intervention effectiveness, articles focusing on informal education, support or advice offered from one parent to another outside of an intervention program were excluded. Study protocols and evidence syntheses were excluded, however if the evidence synthesis matched the review population, intervention and outcome of interest, the reference list was searched for additional primary studies that fit our inclusion criteria.

For the initial search, completed in December 2021, two reviewers (Reviewer 1, Reviewer 2) independently screened a subset of articles (n = 248) from the total number of articles yielded from the search and an inter-rater reliability of 96% (ĸ = 0.80) was achieved. For the second search in September 2024, the process was repeated where two reviewers (Reviewer 3, Reviewer 4) screened a subset of 25 articles and achieved 96% inter-rater reliability (ĸ = 0.78). Given that a Cohen’s kappa score of greater than 0.60 indicates substantial agreement (Cohen, 1960), all reviewers continued to independently screen the titles and abstracts of each retrieved article. For the initial search, reviewers (Reviewer 1, Reviewer 2) independently completed full-text review of each article and reasons for exclusion were recorded. An inter-rater reliability of 97% (ĸ = 0.74) was achieved for full-text screening. Every article was screened by both reviewers (Reviewer 1, Reviewer 2) at the level of title and abstract as well as full-text review. An attempt to find consensus between reviewers (Reviewer 1, Reviewer 2) was first used to address discrepancies in decisions to include or exclude a study. If consensus could not be reached another reviewer (Reviewer 5) was available to adjudicate the decision, this was only required for one article. For the updated search in December 2024, reviewers (Reviewer 3, Reviewer 4) repeated the same process and achieved an inter-rater reliability of 92% (ĸ = 0.80) for full-text screening. Another reviewer (Reviewer 1) was once again available to resolve conflicts but was not required for any articles.

### Data Extraction

The data extraction form was developed by the full research team and piloted by reviewers (Reviewer 1, Reviewer 2) by collaboratively extracting data from one of the included studies. Data from the remaining studies were independently extracted by two reviewers (Reviewer 1, Reviewer 2) in the initial search and two reviewers (Reviewer 3, Reviewer 4) in the updated search. Feedback from parent partners on the data extraction table was obtained to help ensure that meaningful content from the parent perspective was extracted from the included studies. The following data were extracted and recorded in a spreadsheet using Microsoft Excel (Microsoft Corporation, 2016): title, year, author, country, number of participants, parental role (e.g., mother, father, stepparent), number of parent participants, study design, EBD of focus, aims of intervention, intervention outcome measures, parent wellbeing outcome measures, results related to parent wellbeing outcomes. To ensure thorough and consistent extraction of data related to describing the interventions of the included studies, the reporting guidelines outlined by the template for intervention description and replication (TIDieR) checklist was followed (Hoffmann et al., 2014).

### Risk of Bias Assessment

Risk of bias (ROB) assessment was completed by reviewers (Reviewer 1, Reviewer 2) for the initial search and reviewers (Reviewer 3, Reviewer 4) for the updated search using the Cochrane ROB 2.0 (Sterne et al., 2019) or the Risk of Bias in Non-Randomized Studies - Interventions (ROBINS-I) (Sterne et al., 2016) tools matching the corresponding study designs of the included articles. Considering the five domains of bias in the Cochrane ROB 2.0, a judgement of low ROB, some concerns of bias or high ROB in the outcome of interest (i.e., parent wellbeing) was applied. Similarly, a determination of bias in the outcomes measure of interest using the ROBINS-I was achieved by applying one of the following ROB judgement levels to the seven domains of this tool and to the study overall: low, moderate, serious, critical or no information. Initially, ROB assessment for one included study was completed collaboratively by two reviewers (Reviewer 1, Reviewer 2) for the initial search and again by two reviewers (Reviewer 3, Reviewer 4) for the updated search to ensure a shared understanding of the ROB domains. The ROB assessments for all remaining articles were completed individually and reviewers met to come to a consensus to resolve any disagreement. Given the small number of studies yielded through the screening process, no studies were excluded due to risk of bias concerns. The Grading of Recommendations, Assessment, Development and Evaluation (GRADE) (The GRADE Working Group, 2013) approach was applied independently by two reviewers (Reviewer 1, Reviewer 2) for the initial search and repeated for all articles included in the updated search by reviewers (Reviewer 3, Reviewer 4) to determine an overall quality of evidence for the entirety of the data set. The body of evidence in this review was considered in relation to the factors outlined by the GRADE approach that can reduce or increase the overall quality. As indicated in the GRADE handbook, information gathered from the evidence was used alongside reviewer justification, to upgrade or downgrade each GRADE factor by one or two levels depending on how strongly it was determined to influence the quality of the overall body of evidence (The GRADE Working Group, 2013).

### Parent Engagement in Review

Parent partner involvement is reported in alignment with the stages of the review process outlined in the ACTIVE framework of involvement in a systematic review (Pollock et al., 2019). Parent partners were recruited through an open call in a Parent’s Partnering in Research Facebook group. Parent partners contributed to the language of the research question and had critical input in determining inclusion and exclusion criteria, including finalizing our conceptualization of the construct of parent as well as the list of included EBD. Parents also had direct influence on data extraction by identifying content to be extracted. Parent input at the level of the research question, inclusion and exclusion criteria as well as methods for data extraction were reflected in the registered protocol. Parent partners contributed their lens of lived experience to the interpretation of data and development of this manuscript. Lastly, parent partners are included as co-authors on the manuscript and will make use of their established networks to coordinate knowledge dissemination efforts within parent and clinical communities.

### Data Analysis

Given the variability in outcomes measures and a high level of heterogeneity in the populations and peer-led parenting interventions in the included studies a meta-analysis was not completed. Evidence of heterogeneity was examined in data tables presented in the results summarizing features of the included studies. Therefore, a narrative synthesis of the data was completed aligning with the objectives of this systematic review. P-values and mean group differences are presented to report on the intervention effects related to outcomes of parent wellbeing and collated in a table. These metrics were selected as they were consistently reported across the included studies. The range of outcomes related to wellbeing are presented in a table as well the specific outcome measurement tools used to measure these constructs. Peer-led parenting interventions are described in table format in alignment with the TIDieR reporting guidelines (Hoffmann et al., 2014).

## Results

**Figure 1.**
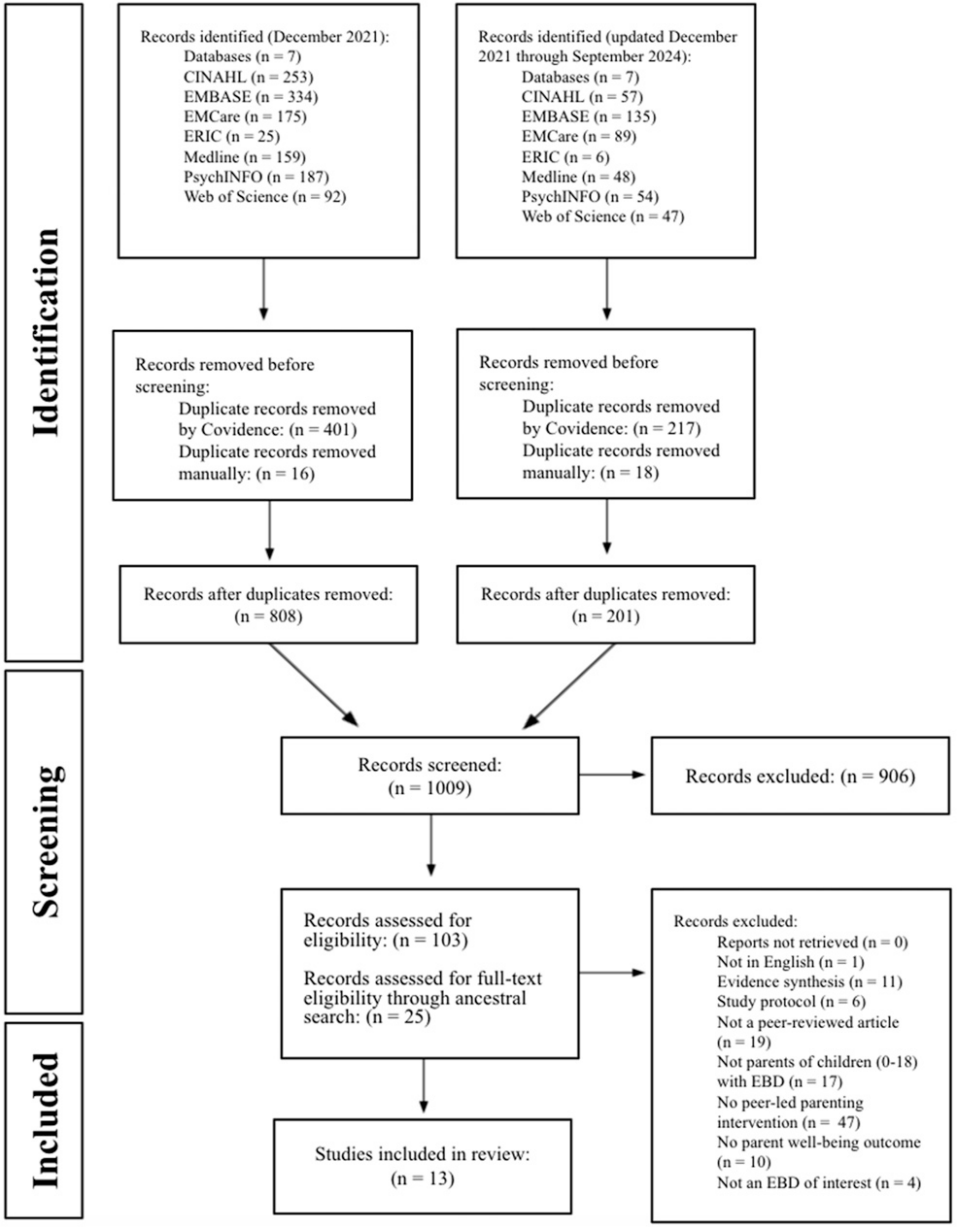
Prisma flow diagram

A systematic search across seven databases yielded ten studies that met the inclusion criteria, and three additional studies were identified for inclusion from searching the references of included articles as illustrated in the PRISMA flowchart (Figure 1). After the initial search, 618 duplicates were removed by Covidence before screening and 1043 articles were screened for eligibility at the title and abstract level. At this level of screening, an additional 34 duplicates were removed manually. A full-text review for eligibility was conducted for 103 articles from the initial database search plus an additional 26 articles as identified through an ancestral search. The thirteen selected articles were published between the years 2011 to 2024.

Across the thirteen included articles, the study designs represented include randomized control trials (n = 4), pilot multisite randomized controlled trials (n = 2), a parallel randomized controlled trial (n = 1), a nonrandomized control trial (n = 1), a pre-post cohort study (n = 1), a pragmatic cohort design (n = 1), a longitudinal follow-up study (n = 1), a mixed methods study (n = 1), and a pilot study (n = 1). Studies were completed in the United States of America (USA) (n = 6), United Kingdom (UK) (n = 4), Australia (n = 1), Canada (n = 1) and Singapore (n = 1). In most studies, participants were primarily mothers (n = 12), however studies also included other participants including fathers (n = 4), adoptive parents (n = 1) and foster parents (n = 1). The EBD populations represented include emotional difficulties (n = 3), mental health conditions (n = 3), generalized behaviour disorder (n = 2), anorexia nervosa (n = 1), generalized emotional or behavioural difficulties (n = 1), aggression, oppositional, or defiant behaviours (n = 1), eating disorders (n = 1), and ADHD/ADD and anxiety/mental health problems (n = 1). Several child-related outcomes were identified across studies, including children’s emotional behavioural problems (n = 4), child mental health problems (n = 4), depression, anxiety, and stress (n = 3), eating disorder symptoms (n = 1), eating disorder assessments (n = 3), academic functioning (n = 1), in-school suspensions (n = 1), child and adolescent psychopathology, (n = 1), and psychosocial impairments due to eating disorder features (n = 1). Almost all studies implemented child and parent outcome measures (n = 12).

Eight peer-led parenting interventions were described in the thirteen included studies: National Alliance of Mental Health (NAMI) basics (Bearman et al., 2022; Jamison et al., 2023), Healthy Mothers Healthy Families (HMHF) (Bourke-Taylor et al., 2024), trauma-informed version of the Incredible Years parenting program (IY) (Conn et al., 2018), Empowering Parents, Empowering Communities (EPEC), encompassing the Being a Parent program (BAP) parenting program (Day et al., 2012a, 2012b, 2022), Experienced Carers Helping Others (ECHO) (Hodsoll et al., 2017), Parent Connectors (PC) (Duppong Hurley et al., 2017; Kutash et al., 2011, 2013), Caregivers-to-Caregivers Training Programme (C2C) (Jiang et al., 2024), and a virtual parent-led peer support group (vPLPSG) (Nicula et al., 2023). Table 1 provides a detailed description of each intervention.

**Table 1.** Summary of Interventions

Intervention name	Parent connectors (PC) (Duppong Hurley et al., 2017; Kutash et al., 2011, 2013)	Empowering parents, empowering communities (EPEC) (Day et al., 2012a, 2012b, 2022)	Experienced carers helping others (ECHO) (Hodsoll et al., 2017)	Incredible years (IY) (Conn et al., 2018)	National alliance on mental illness (NAMI) (Bearman et al., 2022; Jamison et al., 2023)	Virtual parent-led peer support group (vPLPSG) (Nicula et al., 2023)	Healthy mothers healthy families (HMHF) (Bourke-Taylor et al., 2024)	Caregivers-to-caregivers training (C2C) (Jiang et al., 2024)
Rationale	PC is designed to support families of children with emotional disturbances and behavioural difficulties. It utilizes emotional, information, and instrumental support to increase parental engagement in child education and mental health service.	EPEC has 2 components: (1) peer facilitator training (Day et al., 2012a, 2012b), (2) being a parent (BAP) intervention. Using clinical guidelines for treating childhood disruptive behaviours the program incorporates various theories and models. It aims to improve the relationship between a parent and child, decrease behavioural problems, and increase confidence in parenting skills.	The ECHO skill sharing intervention was developed using the medical research council framework for developing complex interventions. It has 3 main components: (1) information about anorexia nervosa with risk and maintaining factors that impact interpersonal function, (2) carer behaviours that maintain illness, (3) increasing carer coping and skills needed for recovery to support behaviour change.	The IY parenting program describes that most parent-child interactions should be positive and preventative rather than disciplinary. This program guides the development and implementation of positive and preventative parenting tools using play. For this study, the curriculum was enhanced by adding information about the impact of childhood trauma on child development and the role of foster parents.	NAMI basics is a family support service led by parent peers for parents of children with mental health concerns. The service is focused on increasing parent education, parent empowerment and introducing problem solving and communication skills.	vPLPSG was created to allow experience sharing and to provide a sense of belonging, capability and advocacy to parents and caregivers of children diagnosed with an eating disorder (ED).	HMHF is a program aimed at supporting the healthy behaviour change of mothers of children with disabilities (inclusive of ADHD/ADD, anxiety and mental health diagnoses).	The caregivers-to-caregivers training programme was created to offer both education and social support to caregivers of people with mental disorders.
Procedures	10 families were assigned to 1 peer-facilitator who engaged in weekly phone calls with participating families throughout the year.	8 topics for group sessions (Day et al., 2012a, 2012b): (1) being a parent; (2) feelings, communication, and culture, (3) play and listening, (4) labels and praise, (5) understanding children’s behaviour, (6) setting boundaries, (7) listening, (8) review and coping with stress. 7 main topics for group sessions in later iteration of the program (Day et al., 2022): (1) parent wellbeing and expectations, (2) understanding children’s needs, (3) emotions and behaviour, (4) child-led play, (5) listening and communication, (6) praise and encouragement, (7) positive discipline strategies.	Carers took a self-directed approach to review the content of the ECHO book, DVD provided. Additional peer-led telephone sessions for guidance were provided.	Foster parent groups and peer-led support outside of groups. The first four sessions are focused on building the foundation for positive parent child relations.	6-Class manualized curriculum that covers: (1) mental disorders and how they affect families, (2) treatment of mental illness, (3) communication skills, (4) managing difficult child behaviours, (5) crisis management, (6) systems of care, and self-care and advocacy.	Each session begins with a parent check-in on the topic of their child’s ED. A presentation led by peer facilitators on a topic that deemed important for caregivers followed.	The program consists of 4 parts: (1) facilitated group workshops, (2) social media group, (3) online module website, (4) e-workbook.	The program is a fully funded education course consisting of 4 modules: (1) introduction to mental health and disorders, (2) medications and adherence, (3) communication, (4) understanding of recovery and resilience.
Intervention provider	Mothers (Kutash et al., 2011) and parents (Duppong Hurley et al., 2017; Kutash et al., 2013) who have been trained in: (1) communication skills, (2) self-disclosure, (3) program mode	Twelve (Day et al., 2012a), 31 (Day et al., 2012b) and 159 (Day et al., 2022) peer facilitators who completed an accredited training program	Eighteen guides, majority had experience of caring for someone with an eating disorder, others were post-graduate psychologists	Two experienced foster parents and one psychologist	NAMI-trained peer parents (Bearman et al., 2022) and family peer advocates (Jamison et al., 2023)	Trained parents of children who experienced childhood ED, treatment, and recovery	7 peer mentor mothers of children with disabilities who completed an accredited training program	Professional psychologists, psychological counselors, and trained peer caregivers
Mode of delivery	Telephone	In-person groups	Telephone	In-person groups	In-person groups	Online platforms	Online platforms	In-person groups
Setting of intervention	Individual telephone sessions (Duppong Hurley et al., 2017; Kutash et al., 2011, 2013)	Schools (Day et al., 2012a, 2012b), children’s centres/schools, (Day et al., 2012a, 2012b), church (Day et al., 2012a), children’s centres (Day et al., 2012b), community centres (Day et al., 2012b, 2022), national health service trusts (Day et al., 2022), and charitable organizations (Day et al., 2022)	Individual telephone sessions	Foster parent groups and individual consult with peer mentor off-site in the community or onsite at a pediatric medical home	Parent group sessions in 5 NAMI affiliates in major cities	Parent group sessions virtually via zoom	Parent groups of 5-20 people virtually via zoom	20-25 caregiver groups in various community location across Singapore
Duration and timing	Once a week for the duration of the school year	8 weekly, 2-h session	Ten, 30- to 60- minute telephone calls	13 weeks, 1.5-h sessions	6 weekly classes, 2.5-h sessions	2-h sessions every 2 weeks for 6 months	3, 2-h sessions over 6 weeks	12 weekly, 2.5-h sessions
Intervention tailoring	Parents could request a call with a peer facilitator as needed	2 groups run for parents who were refugees, 4 groups conducted in French, Arabic, and Somali (Day et al., 2012b)	Guidance phone calls presumably varied between participants	Peer leaders contacted foster parents at least once a week to offer skill implementation support	Service is offered in both English and Spanish	Not reported	Length of time of module interaction was flexible	Caregivers could schedule an appointment at any time for additional support
Planned and actual intervention adherence and fidelity	Planned: - Facilitators tracked call information - Participants completed a facilitator adherence checklist (Kutash et al., 2013) Actual: −70% of participants maintained full contact (Kutash et al., 2011) −89% of participants engaged for at least 60 minutes (Kutash et al., 2013) - Average total time with a peer facilitator over the entire intervention was 305.4 minutes (Kutash et al., 2011) and 370.9 minutes (Kutash et al., 2013) - All participants received at least 60 minutes of phone calls (Duppong Hurley et al., 2017)	Planned: - Peer facilitators attended an accredited training program for a total of 60 hours - Submission of a written portfolio (Day et al., 2012a, 2012b) - Twice monthly supervision of peer facilitators Actual: - Not reported	Planned: - Guides were taught motivational interviewing and trained in behavioural change principles - Guides had regular supervision - session quality was judged for competency in motivational interviewing skills Actual: - Judgement of motivational interviewing was determined to be adequate by those supervising peer carers	Planned: - Peer leaders received 20 hours of training in the IY curriculum - Groups co-led by a IY certified masters-level psychologist - Implemented an IY intervention checklist - Peer leaders received ongoing training for 1-2 hours weekly - Peer leaders spent 5-10 hours weekly preparing, receiving supervision, co-leading groups, and mentoring Actual: - The group leader completed the IY intervention checklist and 74% of prescribed tasks were completed	Planned: - An individual trained in NAMI basics attended a class randomly and completed an adherence checklist to assess prescribed content (Bearman et al., 2022) Actual: - Adherence to the content ranged from 79% to 100% across the various locations and classes (Bearman et al., 2022)	Planned: - Data relating to retention and attendance was collected Actual: - Attendance rate was 60% - Parents attended an average of 7.02 out of 12 sessions	Planned: - All peer facilitators received equivalent training - Delivered content was guided by the same tools for all facilitators - Website was consistent for the duration of the study Actual: - Not reported	Planned: - Not reported Actual: - Not reported

*Note.* This table outlines nine of twelve items described by the Template for Intervention Description and Replication (TIDieR).

PC is a peer-led intervention, developed to decrease stress and improve family coping and engagement (Duppong Hurley et al., 2017; Kutash et al., 2011, 2013). EPEC consists of two components: 1) peer-facilitator training and 2) the BAP parenting course (Day et al., 2012a, 2012b, 2022). BAP is a completely peer-led intervention by parents who have received training to facilitate the program aiming to improve the parent-child relationship, decrease behavioural challenges and increase parenting confidence (Day et al., 2012a, 2012b, 2022). IY uses play to support the development of positive parenting practices and was co-led by a parent peer and a psychologist (Conn et al., 2018). ECHO aims to increase carer coping and skills related to supporting a child with anorexia nervosa and was primarily led by healthcare workers but had a parent coaching component led by peers (Hodsoll et al., 2017). NAMI Basics is run by NAMI-trained parent volunteers focused on increasing parent education and empowerment (Bearman et al., 2022; Jamison et al., 2023). vPLPSG is facilitated by trained peer parents to empower parents of children with eating disorders (Nicula et al., 2023). Similarly, HMHF has multiple online components facilitated by peer mentors aimed at promoting healthy behavioural changes amongst mothers of children with disabilities, including conditions such as anxiety and ADD/ADHD (Bourke-Taylor et al., 2024). C2C offers educational and social support to caregivers and is led by a team of psychologists, psychological counselors, and peer caregivers (Jiang et al., 2024).

While IY (Conn et al., 2018), ECHO (Hodsoll et al., 2017) and C2C (Jiang et al., 2024) are co-led by a parent and a mental health professional, all other interventions were only led by parent peers. (Bearman et al., 2022; Bourke-Taylor et al., 2024; Day et al., 2012a, 2012b, 2022; Duppong Hurley et al., 2017; Jamison et al., 2023; Kutash et al., 2011, 2013; Nicula et al., 2023). EPEC-BAP (Day et al., 2012a, 2012b, 2022), NAMI (Bearman et al., 2022; Jamison et al., 2023), and C2C (Jiang et al., 2024) are community-based interventions. Four interventions were facilitated using phone or online platforms ECHO (Hodsoll et al., 2017), PC (Duppong Hurley et al., 2017; Kutash et al., 2011, 2013), vPLPSG (Nicula et al., 2023) and HMHF (Bourke-Taylor et al., 2024) and one was delivered using a combination of community and home-based delivery (Conn et al., 2018). IY (Conn et al., 2018), EPEC-BAP (Day et al., 2012a, 2012b, 2022), NAMI (Bearman et al., 2022; Jamison et al., 2023), vPLPSG (Nicula et al., 2023), HMHF (Bourke-Taylor et al., 2024), and C2C (Jiang et al., 2024) are group-based interventions. The group-based interventions generally ran 2-3 months in length with weekly meeting opportunities (Bearman et al., 2022; Bourke-Taylor et al., 2024; Conn et al., 2018; Day et al., 2012a, 2012b, 2022; Jamison et al., 2023; Jiang et al., 2024), except vPLPSG, which spanned 6 months (Nicula et al., 2023). ECHO is self-directed in nature with individual opportunities to connect with a peer coach (Hodsoll et al., 2017), and PC (Duppong Hurley et al., 2017; Kutash et al., 2011, 2013) consisted of individual telephone sessions.

Of the thirteen studies included in this review, 7 studies demonstrated a statistically significant difference in outcomes related to parent wellbeing (Bourke-Taylor et al., 2024; Day et al., 2022; Duppong Hurley et al., 2017; Jamison et al., 2023; Jiang et al., 2024; Kutash et al., 2013; Nicula et al., 2023). Six studies reported no statistical significance in parent wellbeing outcomes (Bearman et al., 2022; Conn et al., 2018; Day et al., 2012a, 2012b; Hodsoll et al., 2017; Kutash et al., 2011). Although not statistically significant, these 6 studies reported between-group differences trending toward improved parent wellbeing in the intervention group. Refer to Table 2 for details of statistical results. Measures of stress (e.g., Parenting Stress Index), parent competency and selfcare (e.g., Parent Self-Competence Expectancies Efficacy Knowledge Self-Care), caregiver strain (Caregiver Strain Questionnaire), empowerment (e.g., Family Empowerment Scale), mental wellbeing (e.g., Short Warwick Edinburgh Mental Wellbeing Scale) and depression (e.g., Depression, Stress and Anxiety Scale) are examples of outcomes used as surrogates for parent wellbeing in the included studies. Overall, there was a total of 11 different outcome measures of parent wellbeing that were included in the thirteen studies.

**Table 2.** Study Descriptors and Main Findings of Parent Wellbeing Outcome

Author (Year), country	Aim(s) of the study	Study design	Participants (parental roles, number)	EBD of focus	Parent wellbeing outcome measure	Main finding of parent wellbeing outcome
Kutash et al. (2011), USA	To evaluate the feasibility of implementation of school-based peer-support programs for families of youth in special education programs with emotional disturbances	Randomized control trial	Primarily biological mothers, but grandmothers, fathers, and other relatives also participated (n = 115)	Youth with a special education plan and experiencing emotional difficulties	Family empowerment scale (FES)	No statistically significant difference found in caregiver strain in intervention group (mean score difference = −0.84, *p* = 0.37). No statistically significant difference found in family empowerment (mean score difference = 0.92, *p* = 0.35).
					Caregiver strain questionnaire (CGSQ)	
Day et al. (2012a), UK	To assess the efficacy of a peer-led parenting intervention for socially disadvantaged families	Multisite randomized controlled trial	Primarily mothers, (n = 116)	Generalized behavioural disorder (“disruptive behaviour problems”)	Parenting stress index-short form (PSI-SF)	No statistically significant differences in stress between groups (intention to treat analysis - mean score difference = −4.38, *p* = 0.16; per protocol analysis – mean score difference = −4.70, *p* = 0.17).
Day et al. (2012b), UK	To establish the feasibility of recruiting and training peer facilitators from a socially disadvantaged area; to evaluate the efficacy of a peer-led parenting intervention on parental stress and child behaviour; to identify program acceptability	Pre-post cohort	Primarily mothers, (n = 73)	Generalized behavioural disorder (“parent identified behaviour difficulties”)	Parenting stress index-short form (PSI-SF)	No statistically significant differences in stress before and after intervention (t (28) = 1.7))
						Mean score difference within group before and after intervention = −4.5.
Kutash et al. (2013), USA	To assess the efficacy of the parent connectors (PC) program on caregiver strain and youth emotional wellbeing	Randomized control trial	Primarily biological mothers, but grandmothers, fathers, and other relatives also participated (n = 128)	Students experiencing emotional difficulties	Caregiver strain questionnaire (CGSQ)	Results were statistically significant between groups for reducing caregiver strain (*p* = 0.0387).
Duppong Hurley et al. (2017), USA	To assess the impact attending a peer-support interventions for children with emotional or behavioural disorders on caregiver strain	Randomized control trial	Primarily mothers, but also included other relatives (n = 52)	Youth with a special education plan linked to experiencing emotional difficulties	Caregiver strain questionnaire (CGSQ)	There was a statistically significant difference in caregiver strain (*p* = 0.003).
Hodsoll et al. (2017), UK	To assess the feasibility and acceptability of combining treatment as usual and a carer intervention (experienced caregivers helping others- ECHO); to explore changes in carer and patient wellbeing	Multisite randomized controlled pilot	Primarily mothers of adolescents, (n = 226)	Anorexia nervosa	Depression, stress and anxiety scale - short version (DASS-21)	No statistically significant differences in depression, anxiety, and stress scores between the ECHO group and the ECHO guided group (mean score difference = 0.26, *p* = 0.31). There was also no statistical significance between the ECHO intervention group and the treatment as usual group (mean score difference = 1.64, *p* = 0.72).
Conn et al. (2018), USA	To identify the effect of a trauma-informed foster family parenting program on child behaviour, parental stress, and perceived effect on parenting	Parallel randomized controlled trial	Foster parents, (n = 38)	Generalized emotional or behavioural difficulties (“due to trauma”)	Parenting stress index-short form (PSI-SF)	No statistically significant differences in stress scores within the intervention group (mean score difference = - 6.3, *p* = 0.31), control group (mean score difference = - 3.8, *p* = 0.18), or between groups (mean score difference = −2.5, *p* = 0.84).
Bearman et al. (2022), USA	To assess the efficacy of the national alliance on mental illness (NAMI) basics curriculum	Randomized control trial	Biological mother, biological father, adoptive parent, grandparent (n = 111)	Children experiencing symptoms of a mental health condition before age 13	Parent self-competence expectancies efficacy knowledge self-care (P-SEEKS)	No statistically significant differences measured by PSS (mean intervention group score = 45.06, and mean waitlist group score = 42.70, *p* = 0.30). No statistically significant differences measured by P-SEEKS (mean intervention group score = 19.11, and mean waitlist group score = 19.00, *p* = 0.84).
					Parental stress scale (PSS)	
Day et al. (2022), UK	To assess the efficacy of the being a parent parenting course	Uncontrolled cohort design	Primarily mothers (n = 930)	Youth with childhood behavioral disorders with aggression, oppositional and defiant behaviors	Short Warwick Edinburgh Mental wellbeing scale (SWEMWBS)	A statistically significant difference on wellbeing scale (*p* < 0.001) between time 1 and time 2.
Jamison et al. (2023), USA	To assess the effects of the national alliance on mental illness (NAMI) basics curriculum	Longitudinal follow-up study	Primarily mothers, but other relatives also participated (n = 52)	Children with mental health needs	Parent self-competence expectancies efficacy knowledge self-care (P-SEEKS)	The P-SEEKS measure indicated a statistically significant difference on parent activation and engagement both immediately post-class and 6 months after (*p* < 0.001). There was also a significant decrease in PSS score 6 months after classes (*p* = 0.002), however there was no statistical significance in PSS score immediately post-class (*p* = 0.251).
					Parental stress scale (PSS)	
Nicula et al. (2023), Canada	To assess the feasibility of a virtual peer-support group on parents of children with eating disorders	Convergent mixed methods feasibility	Primarily mothers, but also some fathers and guardians participated (n = 44)	Eating disorders	Eating disorders symptom impact scale (EDSIS), which is framed as a measure of caregiver burden in this study	There was a statistically significant decrease in overall burden score indicated by the EDSIS after 6 months from baseline (*p* = 0.003). However, there was no statistical significance reported 3 months from baseline or between 3 months and 6 months.
Bourke-Taylor et al. (2024), Australia	To evaluate efficacy of group-based workshops led by mothers of children with disabilities	Non-randomized control trial	Mothers (n = 172)	Children with disabilities that could include ADHD/ADD, anxiety/mental health problems	Health promoting activities scale (HPAS), depression anxiety stress scale (DASS 21), positive	Statistically significant increase in HPAS (*p* < 0.001). Statistically significant decrease in DASS 21 score (*p* < 0.001). Statistically significant increase in PGWBI - PWS scores (p = 0.004).
					Wellbeing subscale (PGWBI-PWS)	
Jiang et al. (2024), Singapore	Aims to assess the effectiveness of the caregivers-to-caregivers training programme (C2C) intervention on caregivers of people with mental disorders	Pilot study	Mainly parents (n = 515)	Unspecified mental disorders	Researchers designed their own questionnaire using the delphi method to identify items for outcome measures with the help of psychiatric experts to assess caregiver self-care ability and emotional coping.	There was a statistically significant difference in self-care ability of caregiver’s post-test and at the 2 month follow up (*p* < 0.001). There was also statistically significant improvement in ability to cope with emotions and take care of themselves (*p* < 0.001).

### Risk of Bias and Quality of Evidence

Details about the ROB assessment for each included study across all domains of the ROB tools used can be found in Table 3. One of the included RCTs was deemed to have a high ROB due to limited information about the randomization and allocation concealment process (Conn et al., 2018). Additionally, no information was provided about how missing data were handled, which contributed to a judgement of there being high ROB given the small sample size (n = 38) of this study (Conn et al., 2018). There was some concern for bias noted in all thirteen included studies related to measurement of the outcome given the inherent bias associated with self-reported measures (Bearman et al., 2022; Bourke-Taylor et al., 2024; Conn et al., 2018; Day et al., 2012a, 2012b, 2022; Duppong Hurley et al., 2017; Hodsoll et al., 2017; Jamison et al., 2023; Jiang et al., 2024; Kutash et al., 2011, 2013; Nicula et al., 2023). The understanding that parent wellbeing is likely to always be measured through self-report was taken into consideration and led to a judgement of some concern for ROB instead of high ROB for the measurement of outcome domain. A critical ROB level was applied to the article by Day et al., (2012b) as there was no indication for how confounders were identified or managed as well as a lack of information regarding plans for statistical analysis and the management of missing data. Despite there being a low ROB in relation to randomization procedures, there was determined to be some concerns for bias overall in the remaining studies (Bearman et al., 2022; Bourke-Taylor et al., 2024; Conn et al., 2018; Day et al., 2012a, 2022; Duppong Hurley et al., 2017; Hodsoll et al., 2017; Jamison et al., 2023; Jiang et al., 2024; Kutash et al., 2011, 2013; Nicula et al., 2023) generally due to an inability to compare reported outcomes to a pre-specified plan and large amounts of data missing at follow-up.

**Table 3.** Risk of Bias Assessment

Revised cochrane risk-of-bias tool for randomized trials (RoB2)						
	Randomization process	Deviations from intended interventions	Missing outcome data	Measurement of the outcome	Selection of the reported result	Overall risk of bias
Kutash et al. (2011)	Low risk	Some concerns	Some concerns	Some concerns	Low risk	Some concerns
Day et al. (2012a)	Low risk	Low risk	Low risk	Some concerns	Some concerns	Some concerns
Kutash et al. (2013)	Low risk	Some concerns	Some concerns	Some concerns	Low risk	Some concerns
Duppong Hurley et al. (2017)	Low risk	Some concerns	Some concerns	Some concerns	Low risk	Some concerns
Hodsoll et al. (2017)	Low risk	Low risk	Some concerns	Some concerns	Low risk	Some concerns
Conn et al. (2018)	High risk	High risk	Some concerns	Some concerns	Some concerns	High risk
Bearman et al. (2022)	Low risk	Some concerns	Some concerns	Some concerns	Low risk	Some concerns

Risk of bias in non-randomized studies of interventions (ROBINS-I)								
	Confounding	Selection of participants into the study	Classification of intervention	Deviations from intended interventions	Missing data	Measurement of outcomes	Selection of the reported result	Overall risk of bias
Day et al. (2012b)	No information	Moderate risk	Low risk	Low risk	Critical risk	Moderate risk	Serious risk	Critical risk
Day et al. (2022)	Serious risk	Low risk	Low risk	Low risk	Serious risk	Serious risk	Moderate risk	Serious risk
Bourke-Taylor et al. (2024)	Moderate risk	Low risk	Low risk	Low risk	Serious risk	Serious risk	Low risk	Moderate risk
Jamison et al. (2023)	Serious risk	Low risk	Low risk	Low risk	Serious risk	Serious risk	Low risk	Serious risk
Nicula et al. (2023)	Serious risk	Low risk	Low risk	Low risk	Moderate risk	Serious risk	Low risk	Moderate risk
Jiang et al. (2024)	Serious risk	Low risk	Low risk	Low risk	Serious risk	Serious risk	Low risk	Moderate risk

Results of the ROB assessment helped to determine an overall rating for the quality of the body of evidence this review draws from using the GRADE approach. Table 4 provides details of how this body of literature was rated across the GRADE factors. Due to small samples sizes, the high level of variability in the populations and interventions across the included studies, concerns with ROB associated with study designs, and the need to use surrogate outcomes for parent wellbeing (i.e., stress), this body of evidence can only indirectly answer the research question for this systematic review. Therefore, the quality of the body of evidence was determined to be very low with respect to how it can inform understanding the impact of participation in peer-led interventions on outcomes of parent wellbeing.

**Table 4.** Grading of Recommendations Assessment, Development, and Evaluation (GRADE): Quality of Evidence for Parent Wellbeing Outcome

Factors that can reduce quality of evidence	
Factor	Consequence
Limitations in study design of execution	Downgraded 1 level
Inconsistency of results	Downgraded 1 level
Indirectness of evidence	Downgraded 2 levels
Imprecision	Downgraded 2 levels
Publication bias	None
Factors that can increase the quality of evidence	
Large magnitude of effect	None
Dose-response gradient	None
Effect of plausible residual confounders	None
Overall quality of evidence: Very low	

## Discussion

Findings from this review demonstrate the use of peer-led parenting interventions in the EBD population. This review described eight unique peer-led parenting interventions, with varying levels of peer involvement and across different EBDs. In the included studies, parent wellbeing was represented by eleven different outcomes. Seven studies reported statistically significant improvement in parent wellbeing outcomes. The six studies that did not report statistically significant improvement showed a trend toward improved parent wellbeing on outcome measure scores, which aligns with parent input from qualitative work exploring the impact of participating in peer-led interventions.

In the qualitative results of a systematic review examining peer support for parents of children with chronic conditions, parents identified that peer support provided a sense of belonging and reduced feelings of isolation (Shilling et al., 2013). In studies completed with parents of children with disabilities and challenging behaviour, benefits such as reduced feelings of depression, loneliness and guilt as well as an increased sense of control and confidence were associated with parent peer support (Bray et al., 2017; Dew et al., 2019; Shilling et al., 2013). Some of the benefits associated with peer support relate directly to common conceptualizations of parent wellbeing such as reduced feelings of depression or increased confidence (i.e., self-efficacy), while for others the positive impact on parent wellbeing such as experiencing a sense of belonging can be inferred. The interventions described in this review varied in their format (e.g., in-person, online, telephone), duration and frequency. Further research is needed to explore parents’ perceptions of peer-led parenting interventions in parents of children with EBD and their preferences for format to gain an understanding of what components of the intervention have a positive impact on parent wellbeing.

The concept of parent wellbeing is represented in a broad range of literature including childhood disability and medical complexity (Bourke-Taylor et al., 2021; Bradshaw et al., 2019; Resch et al., 2012), autism spectrum disorder (Giallo et al., 2011; Rutherford et al., 2019) and mental health (Mayberry & Heflinger, 2013; Saed O’leimat et al., 2019). Although the term parent wellbeing has a strong presence in the literature, there is a large amount of heterogeneity in how it is conceptualized and measured as a construct. Some of the constructs used as proxies for parent wellbeing in the child health literature include: stress (Bradshaw et al., 2019; Rutherford et al., 2019), depression (Bradshaw et al., 2019; Giallo et al., 2011; Risi et al., 2021), anxiety (Bradshaw et al., 2019), quality of life (Bradshaw et al., 2019; Rutherford et al., 2019), self-efficacy (Giallo et al., 2011; Rutherford et al., 2019), parenting satisfaction (Rutherford et al., 2019), caregiver strain (Mayberry & Heflinger, 2013) and family functioning (Bradshaw et al., 2019).

The Conceptual Model of Factors Influencing Parent Wellbeing, was developed from work with parents of children with disabilities and provides guidance related to constructs that may influence parent wellbeing (Resch et al., 2012). This dynamic model situates parent wellbeing as being influenced by factors related to the parent or child (e.g., demographics, child’s disability) as well as environmental/social factors (e.g., access to information and services, social and community inclusion) (Resch et al., 2012). The inconsistent findings in improved parent wellbeing may be due, in part, to the challenges in measuring this multi-faceted and dynamic construct. Given the dynamic relationship in the personal and environmental contexts that may shape parent wellbeing further research completed directly with families is needed to define this construct comprehensively and meaningfully.

The parent partners on our research team emphasized the impact of lived experience on what parents perceive as meaningful indicators of wellbeing as well as their perceptions of the value of peer-led interventions. As clinicians and researchers, we need to find ways to authentically represent the influence of lived experience when conceptualizing dynamic constructs such as the wellbeing of parents of children with EBD. We suggest that qualitative, exploration-driven research engaging parents of children with EBD is needed to begin understanding perceptions of wellbeing and the value of peer-led parenting interventions for this population so outcome measures can authentically and accurately measure this construct. Additionally, to enhance the authenticity of conceptualizing parent wellbeing, we advocate for the inclusion of the parent voice in project development. By engaging parents as interest-holders in aspects of research outside of the traditional role related to data collection, the opportunity to develop meaningful research questions, acceptable interventions and relevant results is enhanced by the expert knowledge they contribute through lived experience (Morris et al., 2011; Nguyen et al., 2019). When planning to engage parents in the research process a planned and thoughtful approach is recommended to facilitate meaningful engagement for all parties involved. Co-creation approaches to research have been used in the field of mental health and centre the expertise of individuals with lived experience in all project phases to ensure outputs are meaningful to those most impacted by a problem (Moll et al., 2020; Mulvale et al., 2019). Further, frameworks such as the Involvement Matrix (Smits et al., 2020) exist that can support researchers in planning for research engaging diverse interest-holders such as parents, children, and mental health service providers (Hamilton et al., 2018; Pollock et al., 2019).

## Limitations

A systematic approach to searching and article selection was employed, however, there is a risk that relevant literature was missed or screened out. Although, use of search parameters was minimized to attempt to capture all relevant literature, only peer-reviewed research was included meaning that applicable information in the grey literature (e.g., community parenting program websites, program testimonials by parents) was excluded. Although including only peer-reviewed articles aligns with our research question examining the effectiveness of peer-led parenting interventions on outcomes of parent wellbeing, the exclusion of grey literature restricts analysis to parenting programs with published evidence. This excludes data that may not inform conclusions about intervention effectiveness but could contain clinically relevant insights about this intervention approach. To pilot the data extraction form and ROB assessment, reviewers completed these steps collaboratively for one article for both the initial and updated searches. Despite the small number of articles included, the review team agreed that jointly piloting data extraction and ROB assessment for one article enhanced rigour and reviewer consistency for the remaining studies.

## Conclusions

Peer-led parenting interventions are a model used with the EBD population. Eight different parenting interventions with varying degrees of peer-led components were described in this study. Findings indicate the potential for peer-led parenting interventions to have a positive impact on parent wellbeing. However, given the very low quality of the body of evidence, these findings need to be interpreted with caution. The variation in the findings related to the peer-led parenting interventions described and outcomes measuring parent wellbeing indicate a need for future parent-engaged research to determine how to meaningfully measure parent wellbeing, to better understand parents’ perceived value of peer-led parenting interventions and to determine what aspects of peer-led parenting interventions positively impact parent wellbeing.

## Supplemental Material


Supplemental Material - Examining the Effect of Peer-Led Parenting Interventions on the Well-Being of Parents With Children With an Emotional or Behavioural Disorder – A Systematic Review
Supplemental Material for Examining the Effect of Peer-Led Parenting Interventions on the Well-Being of Parents With Children With an Emotional or Behavioural Disorder – A Systematic Review by Meaghan Reitzel, Kayla Brissette, Ledina Hasanagic, Mona Elmikaty, Lori Letts, Briano Di Rezze, Oksana Hlyva, Anne MacLeod and Michelle Phoenix in Clinical Child Psychology and Psychiatry

## Data Availability

The data supporting the findings of this study are available within the article.*
