# Supplementary material for: Examining the Effect of Peer-Led Parenting Interventions on the Wellbeing of Parents With Children With an Emotional or Behavioural Disorder – A Systematic Review
Source: Clin Child Psychol Psychiatry. 2026 Jan 26;31(2):431–58. doi: 10.1177/13591045261418322 (PMC12992649; doi:10.1177/13591045261418322)
Supplement: Supplemental Material - Examining the Effect of Peer-Led Parenting Interventions on the Well-Being of Parents With Children With an Emotional or Behavioural Disorder – A Systematic Review [file sj-pdf-1-ccp-10.1177_13591045261418322.pdf]

Supplementary Materials

Appendix A - Search Terms

Search Concept			
Parent	Parenting Intervention	Peer Led Intervention	Emotional and Behavioural Disorders (EBD)
Terms			
Parent	Parent training	Peer-led	Depression
Mother	Parenting program	Peer facilitator	Mood disorders
Father	Parenting intervention	Peer mentor	Major depression
Caregiver	Parenting support	Peer-to-peer	Dysthymia
	Parenting education	Peer support	Bipolar disorder
		Peer counseling	Anxiety Disorder
		Peer mediated	Agoraphobia
			Generalized anxiety disorder
			Separation anxiety disorder
			Social anxiety
			Social phobia
			Specific phobia
			Panic disorder
			Posttraumatic stress disorder
			Oppositional defiant disorder
			Obsessive-compulsive disorder
			Attention-deficit/hyperactivity
			Conduct disorder
			Eating disorder
			Behaviour disorder
			Emotional disorder
